# Filling Frail Cavities

**Published:** 1869-01

**Authors:** W. George Beers

**Affiliations:** Montreal


### SELECTED ARTICLES.

### ARTICLE VI.

### *Filling Frail Cavities.*

By W. George Beers, Montreal.

In the variety of cases which come under the observation of the operative dentist, there is none that afford more scope for patient ingenuity and artistic manipulation than filling frail cavities with gold. When in addition to the frequent difficulties of access, excessive flow of saliva, and hyper-sensitiveness, we have frailty and friability of walls in a conspicuous tooth, the steadiest nerve and most delicate manipulation is imperative at every point of progress if the frail walls are to be preserved in their existing integrity.

In this connection we particularly refer to the incisors, cuspids, and bicuspids, as the broadest human grin seldom has more latitude; and the sacrifice of tooth substance for firm borders, beyond the latter teeth, is not only of less consequence for appearance sake, but most often indispensable for the success of gold operations.

It is easier, and to some, more tempting because easier, to extract a frail incisor and replace with an artificial substitute, or to sacrifice tooth substance for firm borders, and perhaps "building out;" but we consider the preserved natural shell of a conspicuous tooth, well filled, infinitely preferable, and more attractive than the most superb specimen of building out ever performed. No one can justly ignore the real science and art of restoring broken angles and fractured crowns with gold, but we are aware of several instances where natural incisors shells, which were capable of preservation, were partially sacrificed to a mania for this operation. Such a tendency should be carefully avoided; for however fashionable the operation may become, nature is superior to art; and a good shell of a conspicuous natural tooth, if possible of preservation, is preferable to any artificial substitute, whether it be of gold or porcelain. Where science and patient taste preside over the manual execution, the results of filling these frail cavities with gold will be more often satisfactory. The danger of fracturing friable enamel when introducing the filling, and the belief that good work cannot be done beside thin borders, leads many to cut away more than is always necessary, but there is a simple way to strengthen the thin walls while filling, and past successes are proof that where such a cavity has ordinary fair play, and where the act of mastication does not bear directly upon the frailty, the shell may nearly always be well filled and preserved, for a sufficient length of time at least, to merit the undertaking. In cases of approximal cavities in the eight teeth particularized, where the walls near the orifice are mere enamel, and the movement of the excavator can be seen through one of the walls, we deem the majority of such cases worth our best efforts to save intact. There are very few patients who prefer losing any angle or part of a natural tooth if it can be saved, even for an indefinite time; and while there are cases of frailty where parts of the enamel are so entirely cracked and jagged that they cannot possibly last out the easiest pressure, we prefer removing any such pieces, and filling out flush, rather than removing the entire wall to get a straight border. Any border, however diagonal, can always be made smooth.

The first principle in filling very frail cavities, after the tooth is prepared, is to adapt a temporary shell of plaster of

Paris, or gutta perch; or in particularly thin cases an impression of the tooth and adjacent parts may be taken and a shield made of cheoplastic metal, as suggested by Prof. Taft, to the natural walls, as a support against pressure, allowing it to extend to the adjoining teeth so as to be immovable during the operation, and so as not to interfere with free access and light. By covering the gum above and behind the teeth, it helps to prevent exudation of saliva. If gutta percha is used, the kind sold for pattern plates for models will not answer as well as the grayish white or brown material used for bougies and catheters, and which while pliable in hot water, and easy of adaptation, is as hard as wood on cooling and may be removed from around the frail wall by a heated plugger.

Smooth margins are as necessary for frail as for firm borders; indeed more so. Sharp edges should be beveled off.

We recently saw a case in point which we successfully filled ten months ago, and which may afford some suggestions for particular cases, though not for all. The case was a superior right cuspid, decayed so extensively on the posterior approximal side as to leave a mere lamina of enamel entirely deprived of dentine on the labial and palatal walls. The patient was averse to extraction, or excision and pivoting, but preferred either to partial excision and building out with gold. A small cavity, the size of a pin's head, had almost worked its way through a natural indentation in the left side of the cusp, into the cavity proper. The nerve was healthy and comparatively well protected. The orifice was larger than the cavity within.

A straight and smooth border was first obtained, and following the rule never to cut into the enamel for retaining points, we excavated at the upper part of the dentine. The hole in the left side of the cusp was then drilled through and enlarged, so as to admit easily a stiff gold wire, four sided and jagged. One point of the wire was then touched with oil and rouge, put through the hole, and pressed upwards until it touched the upper surfaces of the cavity, to the right of the nerve chamber. At the mark left by the rouge a pit was cut, large enough to admit the head of the wire, and deep enough to retain it in place. It was then cut the length required,—sufficiently short to allow of the insertion of the foil plug in the cusp cavity, to render the latter impermeable to fluids. The head of the wire was jagged a little, the better to assist in retaining it in place and was pushed into place. The outward protecting shell of gutta percha, was then moulded around the cuspid and adjoining teeth, covering the cusp cavity as well. When this shell was perfectly hard, the cavity was dried, and the packing commenced by fastening the wire above and below with small pellets of gold. The support of the wire afforded a bridge which compensated to some extent for the loss of dentine at the borders, and consequent absence of any of the usual shaping. Adhesive foil No. 3 was used entirely in thin ribbon strips, the better to carry the layer to the thin walls, and to obtain a greater degree of density. The cavity was thoroughly and tightly packed, without any fracture of the enamel. A very prominent cusp of the lower bicuspid was filed off sufficiently to prevent antagonism in closing: the gutta percha shell was removed and the hole in the cusp filled with a couple of pellets of foil.

The operation was tedious and prolonged, but it has proved a succes so far, and with an ordinary amount of care and cleanliness will, we trust, subserve its object.

We may state *en passant*, that this cavity had been twice filled previous to this operation, and before the cusp hole appeared; the last operation costing $50 in New York.—*Canada Journal of Dental Science.*

---

